# Two-year follow-up of gait and postural control following initiation of recombinant human tripeptidyl intracerebroventricular enzyme replacement therapy in two atypical CLN2 patients

**DOI:** 10.1038/s41598-024-82157-5

**Published:** 2025-01-07

**Authors:** Rahul Soangra, Marybeth Grant-Beuttler, Harriet Chang, Raymond Y. Wang

**Affiliations:** 1 Crean College of Health and Behavioral Sciences, Chapman University, Orange, CA 92866 USA; 2 Fowler School of Engineering, Chapman University, Orange, CA 92866 USA; 3 Department of Physical Therapy, Oregon Institute of Technology, Klamath Falls, OR 97601 USA; 4 Division of Metabolic Disorders, CHOC Children’s Hospital, Orange, CA 92868 USA; 5 Department of Pediatrics, University of California-Irvine School of Medicine, Orange, CA 92868 USA

**Keywords:** Health care, Diagnostic markers, Prognostic markers, Metabolic disorders, Genetics research

## Abstract

Neuronal ceroid lipofuscinosis type 2 (CLN2) is a rapidly progressive neurodegenerative disorder leading to premature mortality. Ambulatory CLN2 patients typically receive standard of care treatment through biweekly intracerebroventricular (ICV) enzyme replacement therapy (ERT) involving recombinant human tripeptidyl peptidase 1, known as cerliponase alfa (Brineura^®^, Biomarin Pharmaceuticals). This study longitudinally assessed the impact of ICV cerliponase alfa ERT on gait, and postural control across a two-year span in two siblings diagnosed with atypical CLN2 disease. Both participants, ID01 (18 years and 8 months old at enrollment) and ID02 (13 years and 3 months old at enrollment), exhibited symptomatic characteristics which were studied longitudinally over three years. Their evaluations assessed postural sway variability, potential for slips and trips, gait metrics, sit-to-stand durations, scores from the sensory organization test (SOT), and gross motor function measure (GMFM) scores. Findings indicated a decline in postural complexity and stability in the medial-lateral (ML) axis, a reduction in toe clearance, and an augmented risk of stumbling for the participants. Over the two-year period of ERT, both siblings exhibited a progressive decline in walking velocity, characterized by reductions in step length and prolonged gait cycle time. The elder sibling demonstrated a notable increase in double support duration, indicative of heightened reliance on proprioceptive input to maintain stability during ambulation. Additionally, sit-to-stand times lengthened for siblings, further reflecting declines in motor function. Despite these challenges, SOT scores showed improvement after two years of ERT, suggesting some preservation of sensory integration. These findings in SOT scores indicate that cerliponase alfa treatment in patients with atypical CLN2 disease may confer benefits in postural stability, lower extremity strength, and ankle stiffness. However, declines in more complex motor functions, including sit-to-stand performance and postural complexity, persist, underscoring the progressive nature of the disease despite ongoing therapeutic intervention.

## Introduction

Neuronal ceroid lipofuscinoses are a group of lysosomal storage disorders; one particular condition, Neuronal Ceroid Lipofuscinosis type 2 (CLN2) disease, ranges in prevalence from 0.22 to 0.90 per 100,000 live births^1–3^. Diagnosis of CLN2 disease is made through demonstration of deficient white blood cell TPP1 enzyme activity and biallelic, pathogenic *TPP1* gene variants^4^. Although the exact TPP1 enzyme substrate is not known, its deficiency leads to accumulation of neuronal intracellular autofluorescent ceroid lipofuscin, followed by neuronal dysfunction, resulting in brain and retinal degeneration^2,5,6^. The clinical signs of CLN2 disease include speech delay, gross motor delay, seizures and ataxia typically evident from 2 to 4 years of age, followed by rapid neurocognitive decline, deterioration in motor and visual abilities, and early death in the first decade of life^7–10^. Subjects with pathogenic variants in *TPP1* which allow for minimal residual TPP1 enzymatic activity may not demonstrate a classical presentation, presenting later in childhood or even adulthood with variable symptoms including epileptic seizures, cerebellar ataxia, dysarthria, or gait disturbance.

Intracerebroventricular (ICV) enzyme replacement therapy (ERT) with cerliponase alfa has been shown to slow the loss of ambulation and verbal skills in symptomatic pediatric CLN2 patients three years of age and older^11–13^. Previous research has used outcomes as motor and language scores of the CLN2 disease Clinical Rating Scale (CRS), an adapted version of the Hamburg Scale for indirect comparison to natural history of 42 untreated controls^11^. ICV administration of cerliponase alfa in atypical CLN2 disease patients may stabilize progression of ataxia and gait deterioration, but long-term follow-up studies are required^14^.

A patient’s motor performance can significantly impact their quality of life, affecting their ability to perform daily activities, participate in leisure activities, and interact with their family and friends. Quality of life for individuals with rare diseases can vary greatly depending on the specific disease condition, its symptoms and severity, available treatments, and ability to perform activities of daily living. Obtaining gait and posture parameters in rare diseases is often challenging due to (i) small patient population sizes with insufficient data to draw meaningful and robust conclusions, (ii) neurocognitive symptoms may make it challenging to collect data, and (iii) participants may find it difficult to follow instructions from the experimenter. In addition, collecting data and communicating with children is associated with several challenges and ethical considerations^15^.

Limited information exists on the impact of CLN2 disease on patients’ fall risk, standing postural instability, and slip and trip risk in the current literature. How the disease affects sensory organization, motor latencies, sit-to-stand times, and Gross Motor Function Measure (GMFM) scores is also unknown. These are essential parameters in assessing the effect of cerliponase alfa treatment upon the well-being and life satisfaction of CLN2 patients. In this study, gait, postural, and mobility measures were investigated on two atypical CLN2 disease siblings. These measures provide insights into the impact of cerliponase alfa treatment on motor performance in patients with CLN2 disease and help understand this rare condition.

## Results

This study presents results of ERT with cerliponase alfa in managing atypical CLN2 disease. We evaluated a comprehensive range of metrics that can strengthen the study’s contribution to understanding and treating CLN2 disease. We did not find any changes in CLN2 rating scale among baseline, year 1 and year 2 scores.

### Gait

**Fig. 1:**
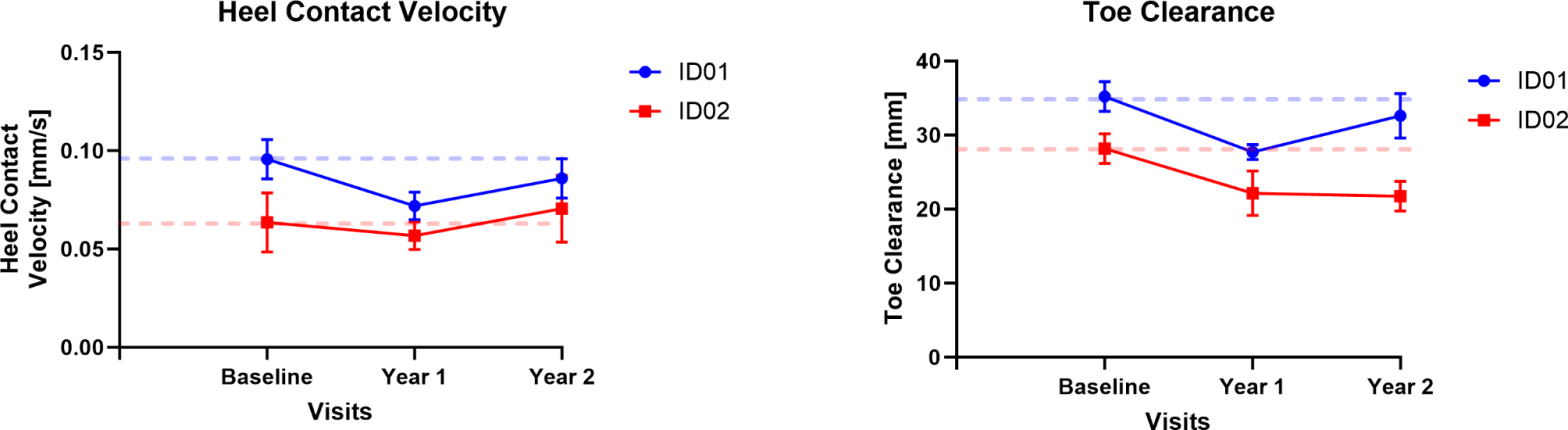
Changes in HCV (left) and Toe Clearance (right) in participants due to treatment for two years. Dotted lines represent baseline values and blue and red represent ID01 and ID02 respectively.

Numerous gait parameters linked to the risks of slipping and tripping were discerned through walking trials (tabulated in Table 1). The patients’ slip risk, as measured by HCV, did not exhibit an increase (as shown in Fig. 1 on the left), but there was a reduction in toe clearance following the baseline assessment suggesting increased risk of tripping (See Fig. 1).

**Fig. 2:**
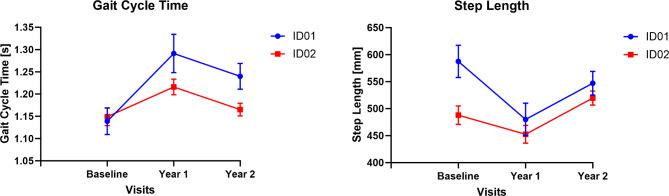
Gait cycle time (GCT) (left) and step length (right) of the participants after 2-year treatment intervention. Where blue and red represent ID01 and ID02 respectively.

We also found the gait speed decreased in siblings during the two years of treatment. Their gait cycle time increased, but step length decreased suggesting increased ataxia and instability with subsequent need for gait compensation (See Fig. 2).

**Fig. 3:**
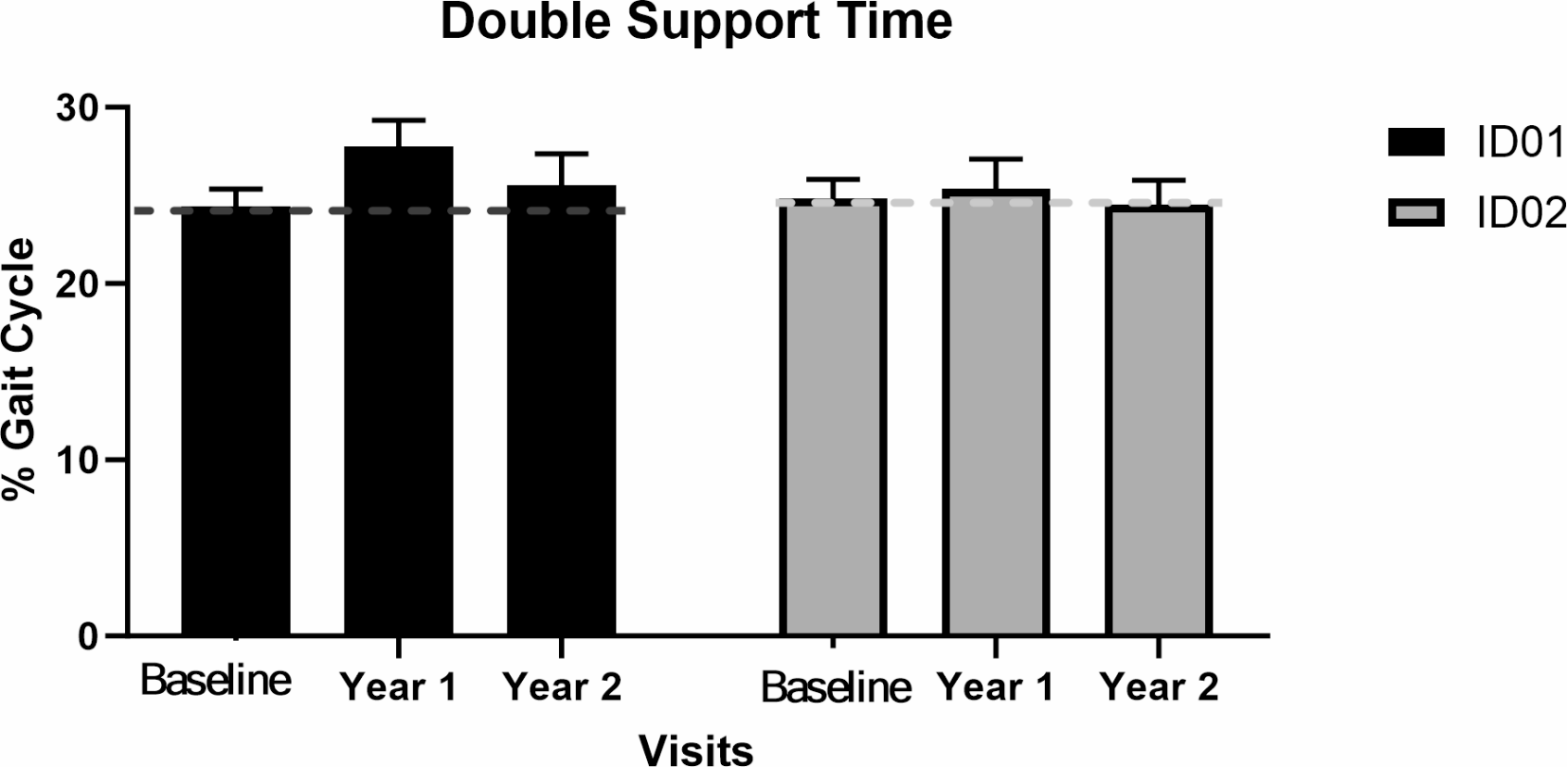
Double support time of the siblings for baseline, year 1 and year 2 assessments. Where dotted lines represent baseline values and black and gray represent ID01 and ID02 respectively.

Gait parameters are tabulated in (Table 1). We found double stance time increased during walking in both participants after intervention suggesting increased ataxia and subsequent requirement for additional sensory input (See Fig. 3). Overall, the gait parameters indicate a cautious walking pattern with increased stability due to longer double support time and shorter steps. However, decreased toe clearance presents a somewhat conflicting risk by increasing the likelihood of tripping.

**Table 1:** Gait parameters for baseline, year 1 and year 2 for ID01 and ID02.

	ID01			ID02		
	Baseline	Year 1	Year 2	Baseline	Year 1	Year 2
Step interval [s]	0.57 ± 0.02	0.64 ± 0.01	0.63 ± 0.01	0.57 ± 0.01	0.60 ± 0.01	0.58 ± 0.004
Step length [mm]	587.5 ± 14.16	480.2 ± 5	547.2 ± 5	488.0 ± 5	452.6 ± 6.98	519.7 ± 0.76
Step width [mm]	199.3 ± 14.78	169.3 ± 4	180.0 ± 3	98.69 ± 3	124.9 ± 8.391	98.42 ± 5.099
Heel contact velocity [mm/s]	0.096 ± 0.007	0.072 ± 0.005	0.086 ± 0.003	0.064 ± 0.001	0.057 ± 0.001	0.071 ± 0.013
gait cycle time [s]	1.13 ± 0.04	1.29 ± 0.03	1.24 ± 0.04	1.14 ± 0.02	1.21 ± 0.01	1.16 ± 0.004
Double support time [s]	0.27 ± 0.01	0.36 ± 0.01	0.32 ± 0.01	0.28 ± 0.01	0.31 ± 0.01	0.28 ± 0.005
Double support time %	24.37 ± 0.38	27.75 ± 0.40	25.56 ± 0.35	24.80 ± 0.37	25.35 ± 0.61	24.46 ± 0.36
Single support time	0.71 ± 0.03	0.82 ± 0.03	0.78 ± 0.02	0.72 ± 0.02	0.76 ± 0.01	0.72 ± 0.002
Single support time %	62.42 ± 0.67	63.90 ± 0.50	63.63 ± 0.30	62.63 ± 0.50	62.62 ± 0.60	62.32 ± 0.05
Swing time	0.857 ± 0.028	0.928 ± 0.03	0.926 ± 0.02	0.859 ± 0.03	0.906 ± 0.012	0.879 ± 0.003
Swing time %	75.27 ± 0.285	71.90 ± 0.3	74.64 ± 0.25	74.77 ± 0.35	74.49 ± 0.20	75.48 ± 0.54
Toe clearance [mm]	35.22 ± 0.66	27.71 ± 0.50	32.64 ± 0.48	28.18 ± 0.44	22.16 ± 0.31	21.74 ± 0.48
Step width Symmetry ratio	1.036 ± 0.001	1.087 ± 0.002	1.049 ± 0.001	1.018 ± 0.001	1.080 ± 0.006	1.132 ± 0.001
Step length symmetry ratio	1.060 ± 0.031	1.032 ± 0.028	1.007 ± 0.012	1.028 ± 0.025	1.035 ± 0.03	0.992 ± 0.03

### Sit-to-stand

**Fig. 4:**
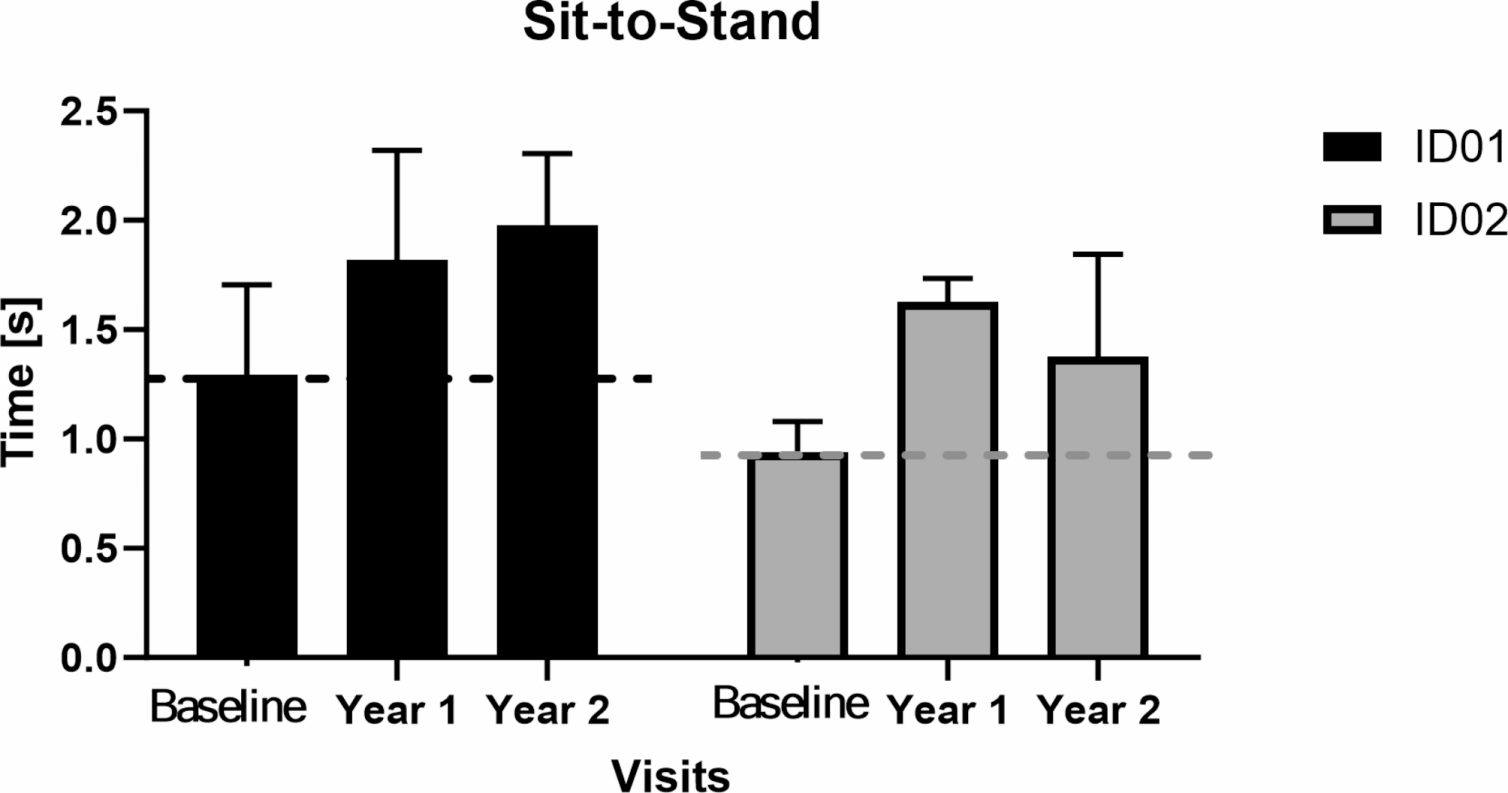
The sit-to-stand times in siblings during baseline, year 1, and year 2. Where dotted lines represent baseline values and black and gray represent ID01 and ID02 respectively.

We also found the STS times increased in both participants (See Fig. 4). We found increased STS times for both siblings suggesting difficulty in transitioning from sitting to standing, indicating potential decline in muscle strength and motor control. This longer duration needed for STS transitions aligns with the progressive nature of CLN2 disease, potentially reflecting its impact on muscular and neuromotor functions.

### Postural stability

**Fig. 5:**
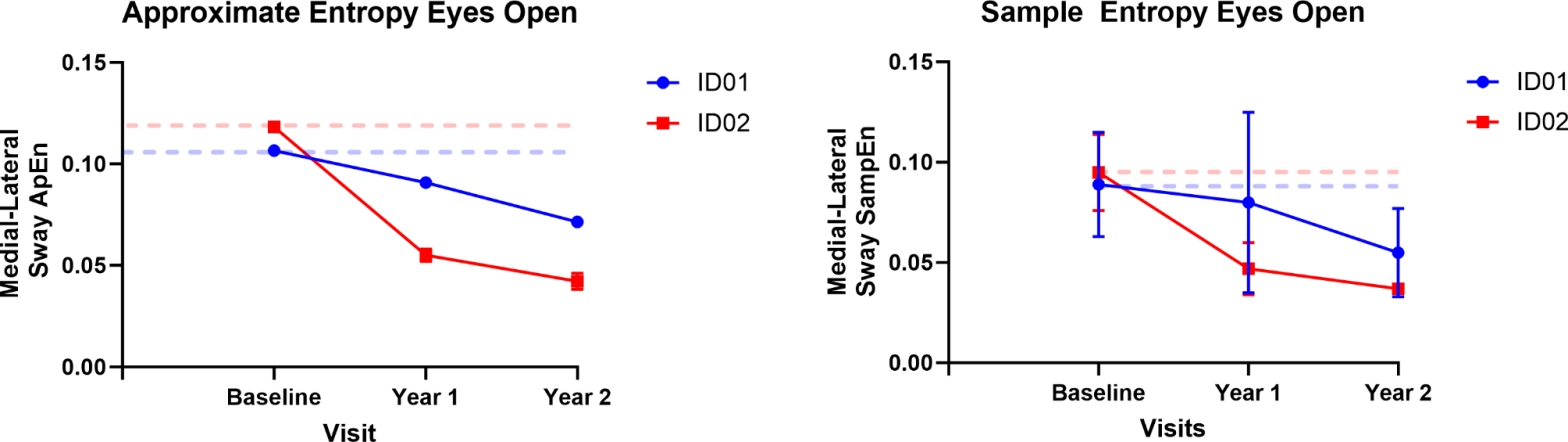
Approximate entropy (left) and sample entropy (right) for ID01 and ID02 in the following two years of infusion. Dotted lines represent baseline values; blue and red lines represent ID01 and ID02, respectively.

We found that ML postural complexity declined in both participants with cerliponase alpha treatment and over two years (See Fig. 5). Approximate entropy (ApEn) declined by 14.7% in year 1 and 32.8% in year 2 for ID01 and decreased by 53.4% in year 1 and 64.3% in year 2 for ID02 from their baseline values. Similarly, sample entropy (SampEn) declined by 10.1% in year 1 and 38.2% in year 2 for ID01 and decreased by 50.5% in year 1 and 61.0% in year 2 for ID02 from their baseline values. The nonlinear postural variability measures are tabulated Table 2 and linear postural variables are provided in (Table 3).

**Table 2:** Nonlinear postural parameters (mean ± standard deviation) for eyes closed (PSEC) and opened (PSEO) condition for ID01 and ID02.Wolf lyapunov exponents, approximate entropy and sample entropy for eyes opened and eyes closed condition are reported for anterior-posterior and medial-lateral sway trajectories.

		ID01				ID02			
		PSEC		PSEO		PSEC		PSEO	
	Visit	AP	ML	AP	ML	AP	ML	AP	ML
Wolf LyE	Baseline	0.73 ± 0.18	0.23 ± 0.09	0.22 ± 0.23	0.46 ± 0.13	1.22 ± 0.05	0.59 ± 0.10	0.57 ± 0.40	0.69 ± 0.29
	Year 1	0.22 ± 0.01	0.18 ± 0.07	0.31 ± 0.26	0.37 ± 0.36	0.52 ± 0.07	0.29 ± 0.02	0.60 ± 0.03	0.11 ± 0.06
	Year 2	1.01 ± 0.26	0.41 ± 0.39	0.47 ± 0.15	0.14 ± 0.05	0.91 ± 0.71	0.21 ± 0.05	0.52 ± 0.13	0.02 ± 0.01
ApEn	Baseline	0.11 ± 0.01	0.09 ± 0.003	0.05 ± 0.01	0.10 ± 0.02	0.16 ± 0.005	0.11 ± 0.01	0.11 ± 0.03	0.11 ± 0.01
	Year 1	0.08 ± 0.01	0.08 ± 0.02	0.04 ± 0.02	0.09 ± 0.03	0.12 ± 0.002	0.08 ± 0.001	0.10 ± 0.006	0.05 ± 0.01
	Year 2	0.15 ± 0.02	0.07 ± 0.03	0.10 ± 0.01	0.07 ± 0.01	0.12 ± 0.05	0.07 ± 0.001	0.12 ± 0.002	0.04 ± 0.004
SampEn	Baseline	0.09 ± 0.01	0.07 ± 0.001	0.05 ± 0.01	0.08 ± 0.01	0.14 ± 0.005	0.09 ± 0.006	0.08 ± 0.03	0.09 ± 0.01
	Year 1	0.06 ± 0.001	0.06 ± 0.01	0.04 ± 0.02	0.08 ± 0.03	0.10 ± 0.001	0.07 ± 0.001	0.09 ± 0.006	0.04 ± 0.009
	Year 2	0.12 ± 0.02	0.05 ± 0.02	0.08 ± 0.01	0.05 ± 0.01	0.10 ± 0.05	0.06 ± 0.004	0.10 ± 0.001	0.03 ± 0.001

**Table 3:** Linear postural parameters for ID01 and ID02 during eyes open and eyes closed conditions. Sway ranges and RMS are reported for anterior-posterior and medial-lateral directions. 95% ellipse are, sway path and dominant median power frequency are reported for ID01 and ID02 patients for baseline, year 1 and year 2.

	Postural variables	PSEC			PSEO		
		Baseline	Year 1	Year 2	Baseline	Year 1	Year 2
ID01	Range AP	21.46 ± 0.63	53.63 ± 0.27	38.65 ± 3.18	26.52 ± 5.84	36.73 ± 14.99	33.34 ± 5.31
	Range ML	8.16 ± 1.275	15.43 ± 1.80	30.05 ± 3.41	7.29 ± 0.09	11.43 ± 5.68	21.94 ± 4.65
	95% Ellipse Area	86.12 ± 3.36	325.3 ± 9.06	588.4 ± 18.2	120.3 ± 4.32	403.9 ± 35.2	371.5 ± 22.3
	Sway Path	530.2 ± 43.10	901.1 ± 7.22	1251 ± 21.5	436.8 ± 19.62	620.6 ± 7.56	831.8 ± 20.1
	RMS_AP	3.96 ± 0.39	8.35 ± 1.06	6.50 ± 0.56	5.17 ± 1.12	9.55 ± 1.43	6.113 ± 0.405
	RMS_ML	1.293 ± 0.120	2.541 ± 1.127	5.450 ± 0.172	1.288 ± 0.077	2.067 ± 1.038	3.304 ± 1.833
	COP_MPF	0.262 ± 0.006	0.244 ± 0.013	0.200 ± 0.000	0.216 ± 0.028	0.205 ± 0.050	0.224 ± 0.034
ID02	Range AP	21.52 ± 3.65	22.79 ± 8.50	64.20 ± 4.16	35.75 ± 6.21	21.87 ± 2.37	20.34 ± 3.82
	Range ML	19.37 ± 4.19	14.54 ± 5.88	40.10 ± 6.16	25.32 ± 1.06	25.97 ± 5.79	23.29 ± 0.72
	95% Ellipse Area	210.9 ± 5.25	195.0 ± 20.5	910.6 ± 76.8	435.7 ± 32.5	378.6 ± 21.7	320.6 ± 18.34
	Sway Path	910.2 ± 15.08	440.3 ± 2.3	1258 ± 23.5	1020 ± 33.8	711.3 ± 13.24	705.7 ± 5.2
	RMS_AP	3.617 ± 0.670	3.966 ± 0.295	6.900 ± 1.536	5.308 ± 1.195	4.461 ± 0.695	4.015 ± 0.054
	RMS_ML	3.366 ± 0.462	2.529 ± 1.761	6.500 ± 1.546	4.537 ± 0.250	5.265 ± 0.348	5.045 ± 0.564
	COP_MPF	0.247 ± 0.012	1.201 ± 0.320	0.200 ± 0.000	0.254 ± 0.041	0.207 ± 0.018	0.213 ± 0.014

**Fig. 6:**
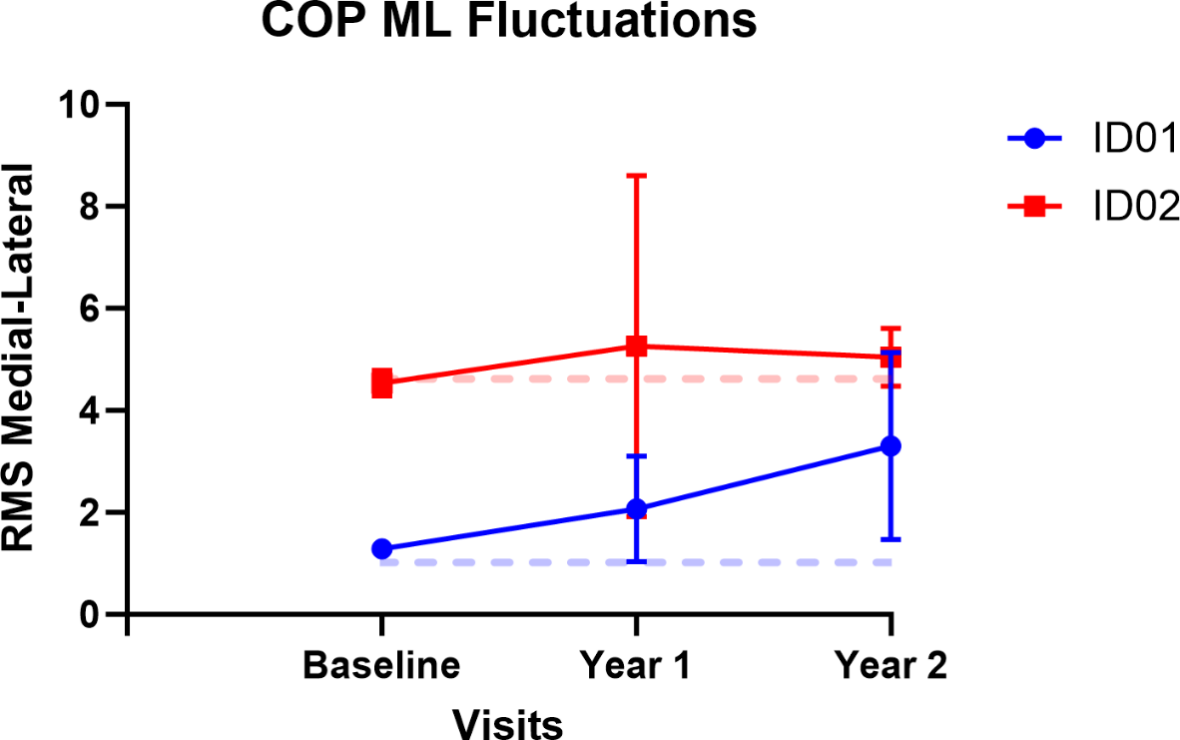
RMS or fluctuations of COP signals in Medial-lateral direction for participants ID01 and ID02. Dotted lines represent baseline values; blue and red lines represent ID01 and ID02, respectively.

**Fig. 7:**
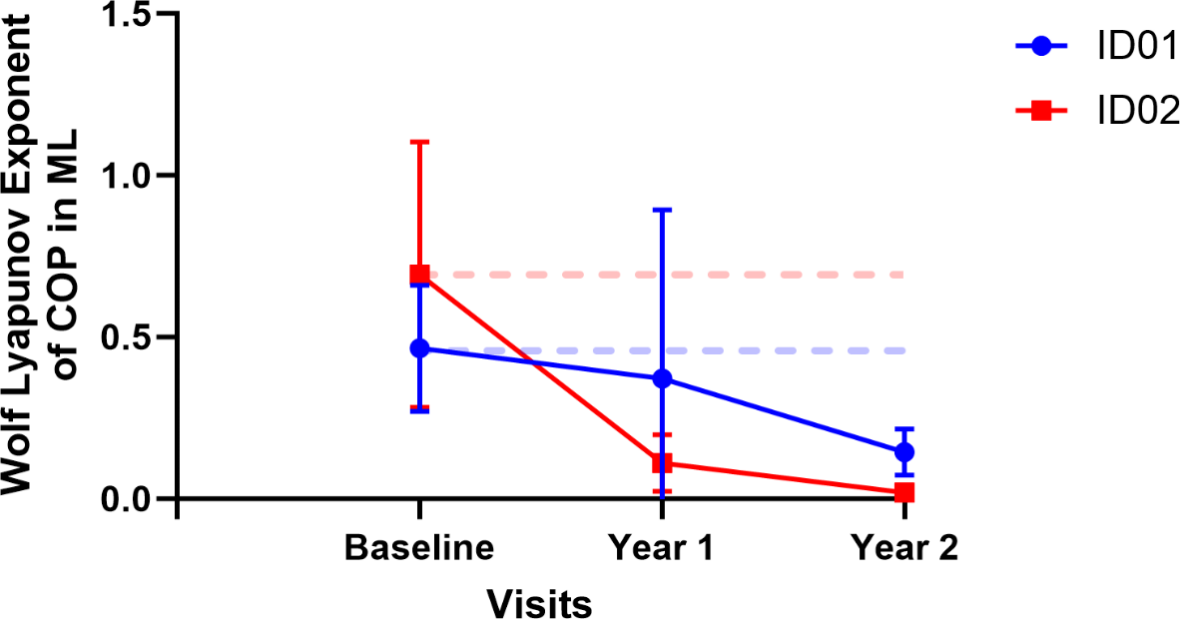
Wolf lyapunov exponents in COP ML directions. Dotted lines represent baseline values; blue and red lines represent ID01 and ID02 respectively.

Additionally, it was observed that linear variability, quantified using the RMS, exhibited an increase in both participants. Conversely, stability, as indicated by LyE, decreased for both participants, as illustrated in Figs. 6 and 7. The RMS values showed a rise of 60.4% in year 1 and 156.5% in year 2 for ID01, and 16% in year 1 and 11.2% in year 2 for ID02. The postural parameters along with measures of linear variability are presented in (Table 3). Furthermore, a decrease in the median power frequency was noted for both participants after the commencement of treatment (See Fig. 7). We noted decreased complexity in postural sway and reduced resilience in postural control (LyE), indicating a decline in the body’s ability to adapt and respond to balance disturbances. Increased signal fluctuations (RMS) in the ML direction suggest compromised postural control, possibly due to impaired proprioception and visual inputs. These results highlight the progressive impact of CLN2 disease on postural stability, reflecting the disease’s detrimental effect on the neuromuscular system and overall balance maintenance.

### Computer dynamic posturography

**Fig. 8:**
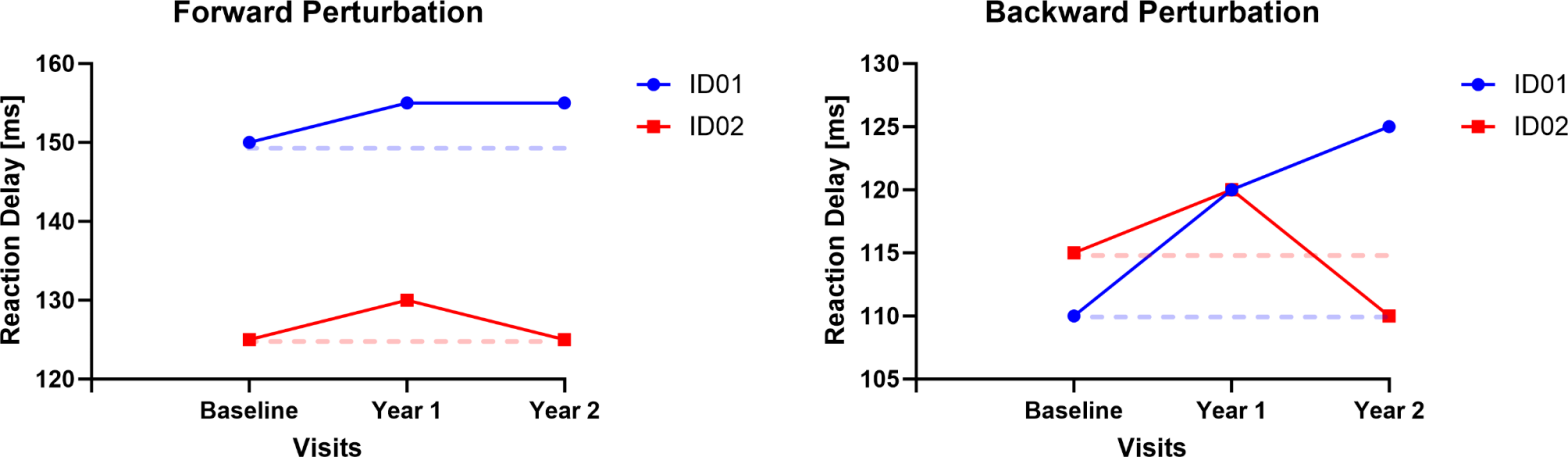
Motor Latencies during forward and backward perturbations. Dotted lines represent baseline values; blue and red represent ID01 and ID02 respectively.

We observed an increase in motor latencies for ID01 in response to both backward and forward perturbations. However, in the case of ID02, motor latencies increased during the first year and decreased during the second year (See Fig. 8).

**Fig. 9:**
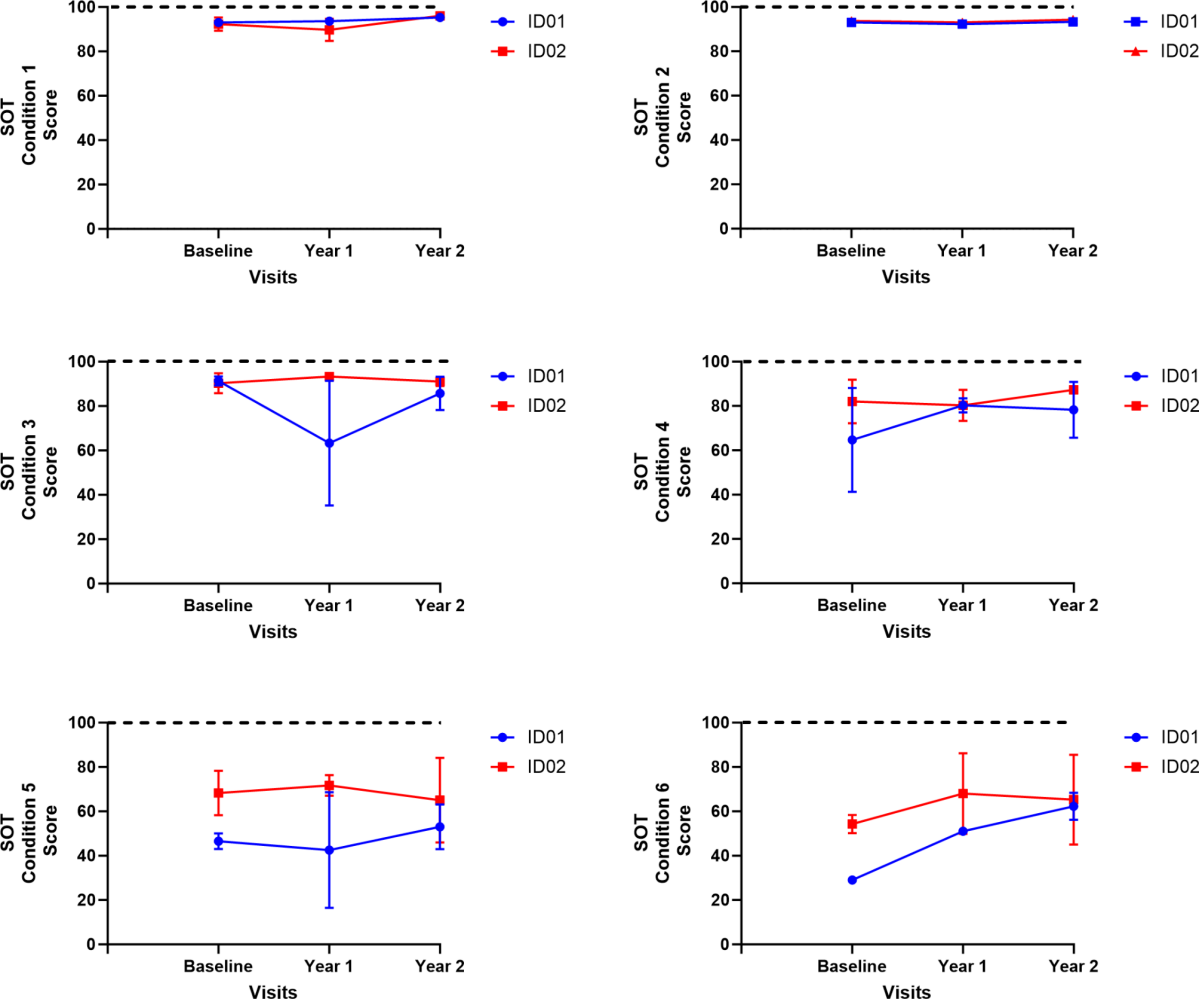
SOT Conditions 1 through 6 and equilibrium scores. Where dotted lines represent highest SOT scores and blue and red represent ID01 and ID02 respectively.

SOT equilibrium scores for all six conditions during baseline, year one, and year two are provided in Fig. 9. We observed stable or improved SOT scores for conditions 5 and 6, indicating maintained or enhanced vestibular function and somatosensory reliance for balance in the siblings. No decline in conditions relying on visual or support surface input was noted, suggesting preserved sensory integration for balance. These findings may indicate that, despite the progression of CLN2 disease, certain aspects of sensory organization and postural control can remain intact or even improve due to growing age or potentially due to compensatory mechanisms or the effects of the ERT intervention.

### Gross motor function scores

**Fig. 10:**
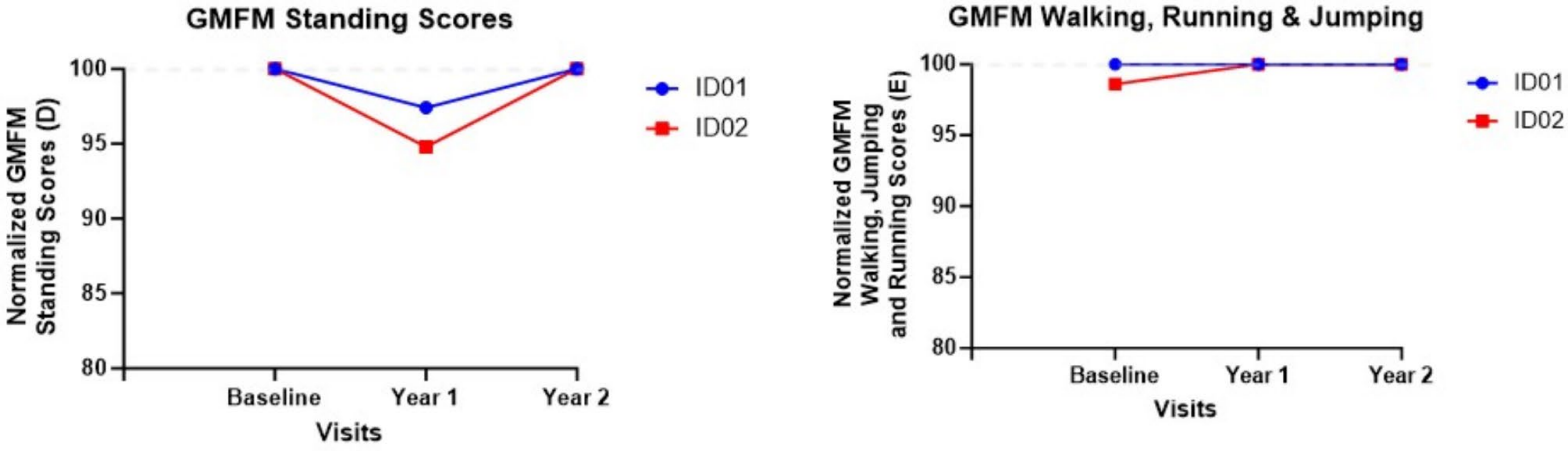
GMFM section D and section E scores during baseline, year 1, and year 2. Dotted lines represent baseline values; blue and red lines represent ID01 and ID02 respectively.

GMFM section D (standing) and section E (walking, running, and jumping) scores are shown in Fig. 10 for baseline, year 2, and year for the siblings. Enhancements in GMFM scores, particularly in the standing, walking, running, and jumping sections, were observed, indicating stabilization or improvement in these motor functions. This improvement contrasts with the typical decline expected in CLN2 disease, suggesting a positive impact of the ERT. The results imply that despite CLN2’s progressive nature, certain motor functions can be preserved or improved with targeted ERT interventions, offering a potential avenue for mitigating the disease’s impact on physical capabilities.

## Discussion

CLN2 is a rapidly progressive neurodegenerative disease, and ERT with cerliponase alfa is administered to slow progression of motor and speech deterioration. Over two years of treatment, we evaluated the effect of treatment upon gait, posture, and fall risk in two siblings who began treatment at different ages, but started intervention together. The sibling study design leverages genetic and environmental similarities to control variability in assessing treatment outcomes. However, careful consideration of baseline phenotypic similarities and the non-linear nature of disease progression is crucial. Classical CLN2 disease typically follows a rapidly progressive course, with children experiencing a decline in motor skills, vision loss, seizures, and cognitive deterioration. Without treatment, the disease can lead to severe disability and a shortened lifespan. Atypical CLN2 disease, on the other hand, presents with a more variable clinical course. While it can still involve the same spectrum of symptoms seen in classical CLN2 disease, the rate of progression may be slower, and the severity of symptoms can vary among affected siblings. Although this sibling study reduces certain variabilities, but a comprehensive validation across broader population is key to establishing definitive cerliponase alfa treatment efficacy.

Our study highlights the complexity of disease progression and the variability in atypical individual responses to the treatment. The observed benefits are intertwined with ongoing challenges in motor control, balance, and the risk of falls, emphasizing the need for a cautious, personalized approach to treatment. We found cerliponase alfa treatment slowed down disease progression as seen through stabilization of SOT and GMFM scores. While the therapy shows promise in stabilizing certain motor functions, it reveals a complex interplay of improvements and persistent challenges in gait and balance. Key gait findings include an increased risk of tripping due to reduced toe clearance, and alterations in gait cycle time and step length suggesting adaptive strategies to maintain stability. The participants took longer durations in STS transitions indicating challenges in force generation and control. Our findings reveal challenges with balance control in CLN2 disease, this is evidenced by increased motor control delays during postural perturbations with elevated RMS fluctuations and decline in complexity in ML direction.

**Fig. 11:**
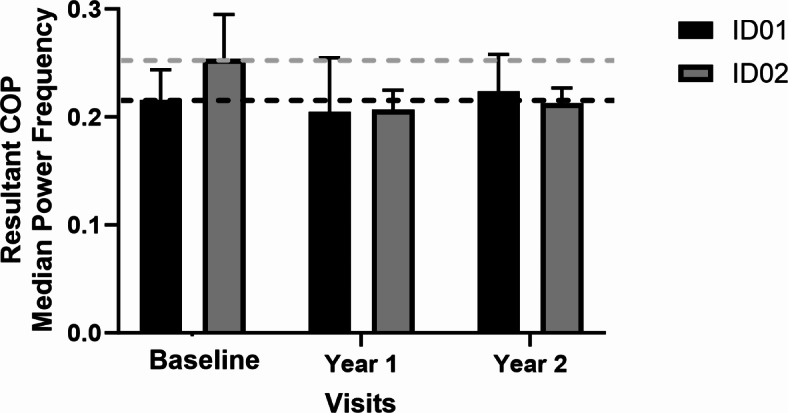
The median power frequency of resultant postural sway signals for ID01 and ID02. Dotted lines represent baseline values; black and gray bars represent ID01 and ID02 respectively.

Utilizing postural analysis, we found that both siblings experienced decreased complexity in their postural sway in the ML direction. For ID01, there was an approximate entropy drop of 14.7% in year 1 and 32.8% in year 2, and a sample entropy decline of 10.1% in year 1 and 38.2% in year 2 compared to baseline values. ID02 showed a larger decrease: 53.4% in year 1 and 64.3% in year 2 for approximate entropy, and 50.5% in year 1 and 61.0% in year 2 for sample entropy (shown in Fig. 5 and Table 2). This reduction suggests a compromised ability to adapt to external ML perturbations, reflecting a decline in motor function. The complexity of postural sway, an indicator of one’s capacity to respond to balance challenges, reveals insights into the robustness of the postural control system. This could be a valuable tool for monitoring the progression of CLN2 disease, as described by O’Keeffe et al.^16^. While complexity exhibited a decrease, we observed a notable rise in signal fluctuations, quantified through the RMS of signals. This increase amounted to an average of 180% in ID01 and 13.6% in ID02 (as demonstrated in Fig. 5). The elevated RMS value in the ML direction indicates a diminished capacity to effectively maintain postural control in this specific orientation, a notion supported by the work of Geurts and coworkers^17^. The amplitude of RMS has been recognized as sensitive to alterations in proprioception^17^ and visual deprivation^17,18^. This observed increase in RMS underscores potential disturbances in proprioception and visual input, contributing to the compromised postural stability in the ML dimension. LyE, assessing postural control resilience^19^, revealed decreased stability for both participants, with declines of 20% and 69.1% for ID01, and 83.9% and 97.2% for ID02 in year 1 and year 2 measurements when compared to their baseline values (See Fig. 7). This trend signifies an ongoing deterioration in the postural stability of the siblings, indicating a reduction in their capacity to generate rapid postural responses. Motor control tests demonstrated delays in response to perturbations, with ID01 showing delays of 5ms (forward) and 12.5ms (backward), and ID02 showing delays of 2.5ms (forward) with no delay (0ms) backward (Fig. 8). Median power frequency reduction in COP signals (Fig. 11) suggests decreased ankle joint stiffness^20^.

Gait parameter analysis revealed a 13.3% rise in gait cycle time (year 1) and 8.8% (year 2) for ID01, and 5.8% (year 1) and 1.4% (year 2) for ID02, indicating decreased walking velocity. A decrease of 12.5% in average step length (ID01) and a marginal 0.38% decrease (ID02) (Fig. 2) contribute to stability by positioning the center of mass closer to the leading foot, reducing the risk of slipping-related falls^21^. Our analysis revealed an increase in double support time (DST) for both participants, with an average 2% increase for ID01 and a marginal 0.1% increase for ID02 relative to their gait cycle times (Fig. 3). Reduced duration of the stance phase during the gait cycle, influenced by proprioceptive input, may adversely impact lower extremity motor control during walking^22^.

The escalation of horizontal heel contact velocity has been linked to a higher susceptibility to falls caused by slipping incidents^23^ and it remained unchanged during the intervention among CLN2 siblings. However, we observed a decrease in toe clearance, amounting to an average reduction of 5 mm in ID01 and 6.2 mm in ID02 (See Fig. 1). This decrease in toe clearance accentuates the risk of tripping for the siblings, thereby increasing their vulnerability to such incidents.

STS times noticeably increased for both participants, with ID01 showing increments of 525 ms (year 1) and 680 ms (year 2), and ID02 displaying increases of 685 ms (year 1) and 435 ms (year 2) (Fig. 4). Longer STS times suggest challenges in generating force and control during transitions, indicating potential reductions in muscle strength and overall physical capacity.

Upon analyzing for sensory integration, we observed SOT equilibrium scores for conditions 5 and 6 either increased or remained unchanged, indicating the absence of vestibular dysfunction in the siblings^24^. Notably, there was no decrease observed in conditions 4 to 6, which point towards a reliance on support surface or somatosensory inputs to sustain balance. Similarly, there was no decline in conditions 2 to 3 and 5 to 6, suggesting that there was no significant visual reliance or preference among the siblings. Since vision loss is a common symptom of CLN2 disease, and it can greatly affect a patient’s quality of life. We noticed ID01 is currently facing significant vision-related challenges, and these difficulties are greatly affecting his ability to move around. These findings strongly suggest that the sensory organization skills, guided by central adaptive mechanisms for maintaining balance, were either maintained or possibly even improved during the course of the ERT intervention or with the advancement of age (as illustrated in Fig. 9).

Following a two-year period of ERT intervention, both siblings displayed enhancements in their GMFM scores, indicating recovery or overall improvement compared to baseline. Notably, GMFM section D (standing) and section E (walking, running, and jumping) scores either improved or remained stable during the treatment period, suggesting potential stabilization of performance in these areas (Fig. 10). While our findings have substantial implications for functionally assessing the clinical trajectory of CLN2 disease in terms of gait and posture metrics, it is noteworthy that certain parameters could detect nuanced deteriorations attributed to CLN2 disease. Illustratively, parameters such as STS duration, ML postural complexity, stability (LyE), motor control delays during perturbation (both forward and backward), sway median power frequency, DST times, and gait velocity exhibited the potential to discern subtle declines associated with the advancement of CLN2 disease.

While our findings indicate that cerliponase alfa therapy provides benefits in stabilizing postural control and reducing ankle stiffness, specific measures such as sit-to-stand times, postural complexity, and toe clearance displayed a decline over the treatment period. This decline in sit-to-stand performance and postural complexity likely reflects the progressive neuromotor impairments inherent to atypical CLN2, which may continue to affect motor tasks that require complex coordination and strength despite some therapeutic benefit. The reduction in toe clearance, leading to increased tripping risk, highlights these persistent challenges in neuromotor function. Thus, while cerliponase alfa appears to support certain aspects of motor function, the progression of the disease continues to impact more complex motor tasks, necessitating careful interpretation of the therapy’s efficacy in the context of the disease’s natural course.

Conversely, assessments such as SOT scores, GMFM scores exhibited negligible alterations due to the deceleration in disease progression. It is noteworthy that our outcomes are divergent from the previous conclusions drawn by Schaefers^25^, who reported a reduction in GMFM-88 scores in CLN2 patients. Nonetheless, this discrepancy might be attributed to our specific evaluation of GMFM subsections D and E, which might not possess adequate sensitivity to detect the subtle progression of the disease over the stipulated 2-year timeframe. In addition to the specific evaluation of GMFM subsections D and E, the differences in outcomes between our study and Schaefers’ report may also be attributable to the younger age and typical presentation of CLN2 in their cohort, compared to the older, atypical presentation in our participants.

Some of our study limitations include a small sample size of only two siblings, and lack of a control group to firmly attribute effects to the treatment. In addition, the inherent variability of atypical CLN2 disease complicates the interpretation of treatment outcomes. Although the strength of our sibling study with similar genetic and environmental commonalities to minimize variability is a two-year follow-up but this duration may not be sufficient to fully capture the long-term effects of the therapy. As a potential avenue for further research, a study encompassing a more extensive participant cohort could substantially contribute to the generalizability of the observed gait and postural metrics, as well as provide insights into their correlation with the progression of CLN2 disease.

In summary, our findings highlight the significant impact of CLN2 disease on gait and posture, alongside evidence suggesting that cerliponase alfa may decelerate certain aspects of disease progression, such as postural stability, as indicated by improvements in SOT scores. However, ongoing deterioration was noted in metrics like ML instability, STS durations, and other gait parameters, underscoring the progressive nature of atypical CLN2 despite therapeutic intervention. This observation is consistent with the slowed, but ongoing deterioration in clinical severity scoring on long-term follow-up of classical CLN2 subjects^12^. The inherent variability of atypical CLN2, coupled with the absence of pre-treatment kinematic data and longitudinal control data, limits our ability to attribute these changes exclusively to ERT. Consequently, while our results point to a potential therapeutic role for cerliponase alfa, they also suggest that both the disease’s natural progression and ERT may contribute to the observed outcomes in these two siblings. Further research with a larger cohort and comprehensive baseline assessments will be crucial to accurately delineate the specific effects of ERT and to refine gait and posture metrics as reliable markers for tracking disease stages and progression in CLN2.

## Methods

### Human subjects

The planning, conduct, and reporting of this project are in accordance with the Helsinki Declaration as revised in 2013. The study was approved by the Children’s Hospital of Orange County Institutional Review Board (IRB#190219). Written informed consent was obtained from subject ID01 and parents of subject ID02; subject ID02 provided assent to participate in the study. ID01 is currently at age of 22.4 years, and carries mutations in the TPP1 gene, specifically c.837 C > G and c.508 + 4 A > G. Early in life, at 5 years old, the participant exhibited learning difficulties and clumsiness, leading to a diagnosis of autism spectrum disorder at the age of 13. As the participant aged developed a progressive tremor, ataxia, cognitive decline, and retinopathy. Treatment with Brineura was initiated when he was 18 years and 9 months old.

**Fig. 12:**
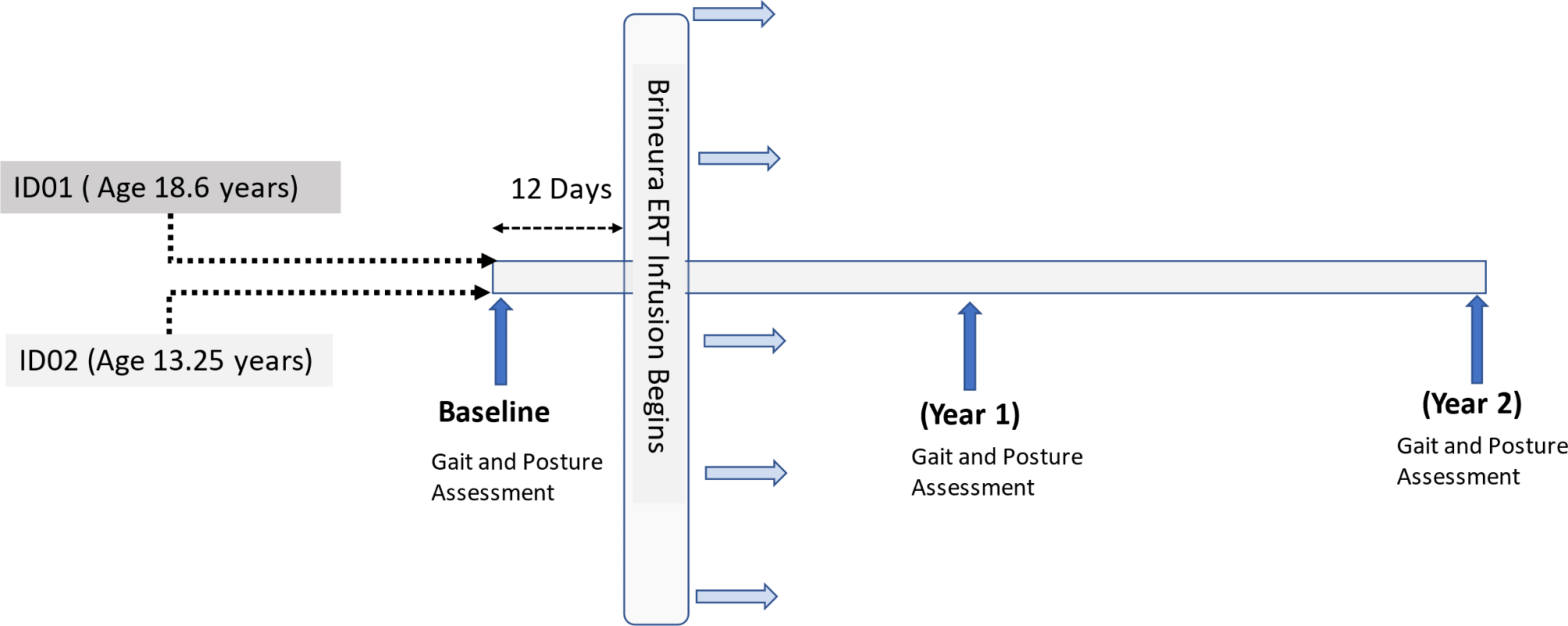
The timeline of assessments during baseline and Years 1 and 2.

ID02 is currently 17.75 years old, also possesses the TPP1 gene mutations c.837 C > G and c.508 + 4 A > G. In her early childhood, around 5 years of age, she was marked by learning and feeding difficulties. These initial symptoms were later accompanied by a mild tremor, ataxia, and cognitive impairments. She began receiving Brineura treatment at the age of 14 years and 1 month. The medical history details are provided in Table 4. The treating physician quantitively assessed disease progression using CLN2 clinical rating scales. Both siblings had pathogenic mutations on both alleles of the CLN2 disease. A schematic of how the study was structured is detailed in (Fig. 12).

**Table 4:** Concise overview of medical history, symptoms, genetic findings and other relevant information for ID01 and ID02.

Attribute	ID01	ID02
Age (during study initiation)	19 years	15 years
Initial symptoms	Memory difficulties, decline in social skills and learning, clumsiness, poor coordination, dyslexia	Failure to thrive (FTT), cyclic vomiting, neutropenia, learning difficulties
Subsequent symptoms	Headaches, vision difficulties, tremors, concussion effects, swallowing difficulties, speech and balance issues, cerebellar atrophy	Difficulty in school, fatigue, shaking episodes, slight right hand tremor
Family history	ADD in brother, FTT and possible developmental delays in sister, migraines in mother	CLN2 disease in older brother, ADD in another brother, migraines and fibromyalgia in mother
Genetic findings	Two mutations in TPP1 gene (c.837 C > G / c.508 + 4 A > G)	TPP1 gene mutations c.837 C > G and c.508 + 4 A > G
Treatment	Recombinant human TPP1 enzyme infusions (cerliponase alfa; Brineura)	Recombinant human TPP1 enzyme infusions (cerliponase alfa; Brineura)
CLN2 scoring (2020)	Ambulation: 2 of 3, Speech: 3 of 3	Ambulation: 3 of 3, Speech: 3 of 3
CLN2 scoring (2022)	Ambulation: 2 of 3, Speech: 2 of 3	Ambulation: 3 of 3, Speech: 3 of 3
Intervention changes	Switch from skull-implanted Rickham reservoir to chest port, neurocognitive testing, ophthalmologic evaluation, MRI, gait assessment, CSF sampling	Switch from skull-implanted Rickham reservoir to chest port, neurocognitive testing, ophthalmologic evaluation, MRI, gait assessment, CSF sampling
Birth history	Born at 37 weeks, jaundice treated with home phototherapy	Born at 37 weeks, NICU stay for 5 days due to hyperbilirubinemia, required phototherapy
Review of systems	Headache, tremors, difficulty swallowing, change in speech quality, no seizures	Difficulty with weight gain, vomiting, no unsteadiness/falls

### Gait and posture assessments

Participants were asked to perform (i) overground walking, (ii) instrumented treadmill walking, (iii) postural standing for 1 min, (iv) sit-to-stand, (v) GMFM-88 scores for standing (section D) and walking, running, and jumping (section E). The gait and posture measurement protocols consisted of the following tests:

#### Overground walking

CodaMotion 3-D Analysis System (Charnwood Dynamics Ltd., Leicestershire, UK) with four CODA optical sensors was used for overground walking data collection. The motion analysis system captured body segments’ vertical, horizontal, and rotational movements by tracking the attached active marker positions. The infra-red light-emitting diode markers were attached to bony anatomical landmarks at the skin. A cluster of four markers were placed at each segment (thigh, shank, upper arm, and pelvis). A total of 22 markers were placed bilaterally at the following sites: the 5th metatarsal head, the base of the 5th metatarsal, with clusters placed on the shank, thigh, and pelvis. Clusters were used to mark virtual markers at the anterior superior iliac spine (ASIS), femoral head, lateral and medial femoral epicondyle, lateral tibial epicondyle, and medial and lateral malleoli. The Ground Reaction Forces (GRF) data were collected using two forceplates model Bertec BP400600 (Bertec, Columbus, Ohio 43219) embedded in the 15 m long and 4 m wide walkway. The participants were asked to perform walking on a 10 m-long walkway at their preferred pace.

#### Treadmill walking

In addition to overground walking, participants performed at least two 1-minute walking trials on the treadmill. All participants were asked to continue walking while looking at the static reference colored object and maintain their walking speed. A total of 4 infra-red reflective markers were placed on the heels and toes of both feet. All participants were harnessed for safety while walking on the on GRAIL (Gait Real-Time Analysis and Interactive Lab, Caren, Motek Medical, Netherlands). All participants were asked to walk at their preferred walking speed. The protocol for speed determination was kept the same for both participants^26^. The GRF was filtered and normalized prior to evaluating the walking and Sit-to-Stand (STS) dynamics. The whole-body kinematics and GRFs were collected at 100 Hz and 1000 Hz, respectively, and then low-pass filtered at 6 Hz cut-off frequency (4th order, zero-lag Butterworth).

#### Assessment of slip and trip risk among participants

*Heel contact velocity (HCV)* is an instantaneous velocity at the heel contact phase of gait. *Toe Clearance*: The minimum toe clearance is the vertical distance by which the toe clears the ground during the mid-swing phase.

#### Gait parameters

*Gait cycle time (GCT)* is the time to complete one gait cycle. The gait cycle starts when the one-foot heel strikes the ground and ends when the same foot contacts the ground again. *Step Length* is the distance between the heel contact of one foot and the point of heel contact of the opposite foot. *Double Support Time (DST)* is when both feet touch the ground during walking. *Single support Time (SST)* is when one foot is in contact with the ground during walking.

#### Sit-to-stand (STS) protocol

**Fig. 13:**
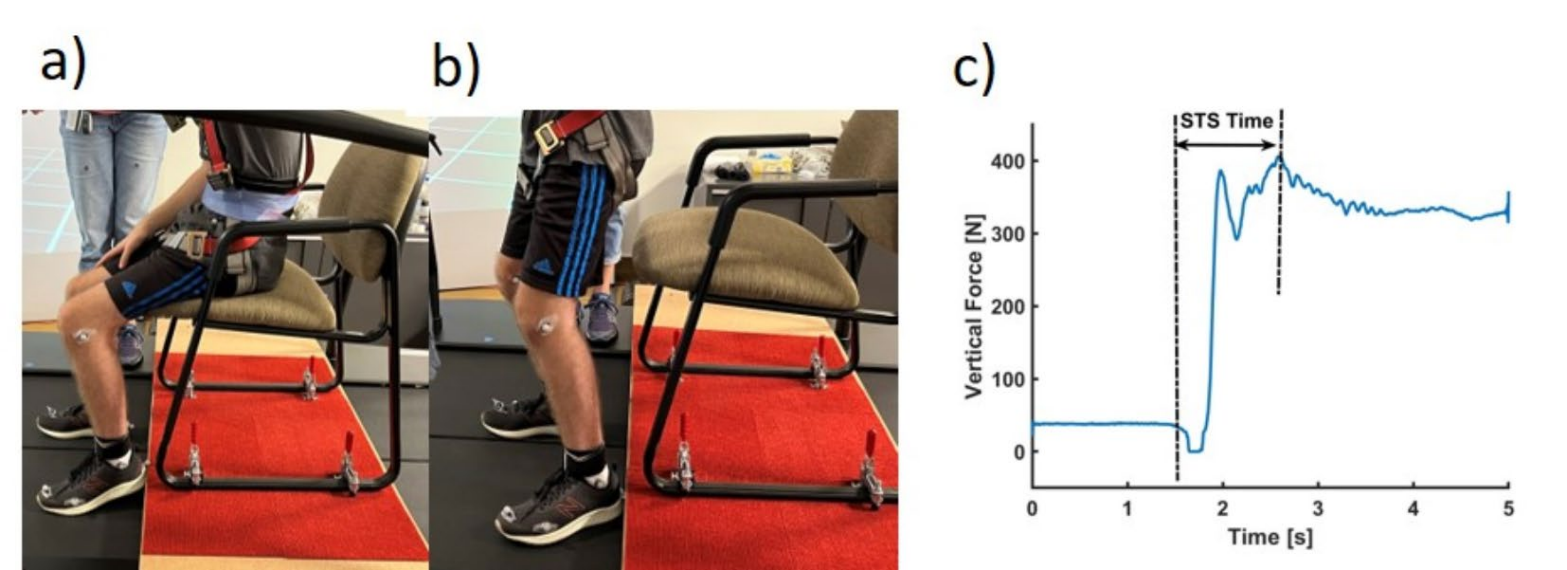
(**a**) Sitting position, (**b**) standing position and (**c**) vertical force for Sit-to-stand evaluation.

Understanding the abilities of CLN2 patients to complete the everyday task can indicate the quality of life and functional limitations. STS is a fundamental activity of daily living and can provide insights on the health status of CLN2 patients and potential effects of cerliponase treatment. STS starts with a static position followed by dynamic activity leading to an upright posture^27^. The dynamics of STS are usually studied using kinematic and kinetic variables. In this study, we evaluated STS times from the forceplate vertical forces (See Fig. 13). STS is generally a symmetrical activity in healthy individuals^27,28^. STS movement requires greater muscle strength than other daily activities, such as walking or stair climbing^29^. STS times have been associated with risk of falls^30^, adverse health-related events, and hospitalization^31^.

The participants sat comfortably on a chair with a backrest and armrest, were asked to keep their thighs and feet parallel, and were instructed to use armrest/knee for support while performing STS task. The chair was held on a platform near the force plate, such that the feet of participants rested on the forceplate during standing. The spacing between feet was maintained at 15 cm. Chair popliteal height was 45 cm, and knee angle was maintained from 85°–90°. Participants were instructed to sit such that the thigh and buttocks rested on the chair and did not use backrest of the chair. Participants were asked to wait for an auditory signal before initiating movement. The data was recorded for at least 15 s in total. The participants were given the auditory signal to stand after at least 3 s of data collection to ensure sitting, postural transition, and stabilized standing were collected in all trials. Each participant performed at least three STS trials. Figure 13 shows sit-to-stand performance (2a-2b) and extraction of STS times from vertical forces (2c).

#### Postural stability

Standing is a complex postural task dependent on numerous sensory inputs across multiple temporal-spatial scales. Standing or postural control depends on integrating multiple sensory inputs, spinal and supraspinal circuits, cognitive functions, peripheral neuromuscular systems all operating at different time scales^32^. Thus, postural control is inherently nonlinear and the relationship between sensory inputs^33^ and muscular outputs is continuously modulated over time^34^.

Participants were instructed to stand still for 1 min with their eyes open (PSEO) facing a target 4 m away and 1 min with their eyes closed (PSEC). Participants were instructed to keep their feet shoulder-width apart on the two adjacent forceplates. Postural sway measures such as range, 95% ellipse area, sway path, root mean square (RMS) and dominant median power frequency were evaluated. In addition, nonlinear variability was assessed through postural sway complexity such as approximate entropy, sample entropy and Wolf’s Lyapunov exponents.

#### Variability of postural sway

Diminished complexity of postural sway has been associated with a reduced ability to adapt to stressors. We evaluated postural sway complexity during standing using approximate and sample entropy techniques. These are some of the nonlinear time-series analysis techniques^35^ that have been used to quantify the degree of re-occurrence of repetitive patterns in medial-lateral (ML) fluctuations.

#### Root mean square

 This measures the standard deviation of the displacement of center of pressure (COP) in the ML direction and can quantify average absolute displacement around the mean COP.

#### Lyapunov exponents (LyE)

We computed resultant COP trajectories during the three visits and evaluated Wolf’s Lyapunov Exponent^36^. LyE is an important chaos tool to determine dynamic stability. LyE quantifies the divergence rate of nearby trajectories in state space^37,38^. It measures the ability to resist postural perturbations to maintain balance. A higher LyE means a higher capacity of the postural control system to respond quickly and flexibly to different postural stimuli^19^. LyE estimates the orbital divergence of 1D time series data of COP trajectories.

#### Postural sway frequency

Postural sway in humans has two fundamental components (i) a slow non-oscillatory component and (ii) a faster damped-oscillatory component. Postural control results from a feedback loop, where the sensory systems detect body sway and appropriate motor responses are generated to stabilize the body’s orientation. The damped oscillatory’s mechanistic source is an inverted pendulum’s feedback control^39^. The resultants of the COP trajectories were extracted, and Fast Fourier Transform (FFT) was used to evaluate the median power frequency. The median power frequency is an index of ankle stiffness; the higher frequency of postural sway indicates higher ankle joint stiffness^20^.

#### Computer dynamic posturography (CDP)

NeuroCom^®^ SMART Balance Master (NeuroCom, Natus, CA, USA) was used for computer dynamic posturography. It contains two forceplates with a visual surround. Sensory Organization Test (SOT) and Motor Control Test (MCT) assessments were conducted. During the testing protocol, the participants were asked to put their bare feet parallel on the forceplate while looking straight ahead. An experienced physical therapist conducted CDP.

#### Sensory organization test (SOT)

**Fig. 14:**
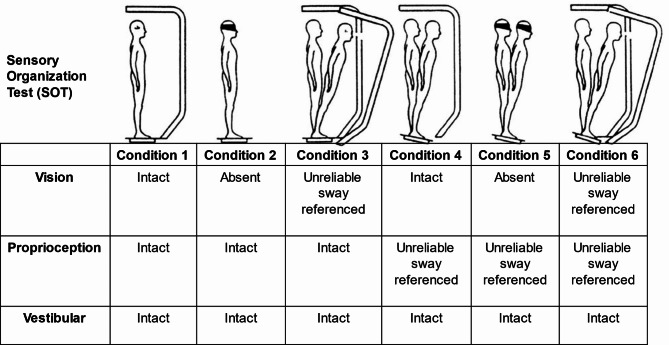
The six testing conditions of SOT. The first three conditions comprising condition 1–3 (eyes opened, eyes closed and sway referenced) involve a fixed platform. The last three conditions condition 4–6, involve a sway referenced platform.

This test quantifies the ability of participants to integrate and process sensory inputs to maintain balance in single or combined conditions of eye-opening condition (eyes open versus eyes closed), the sway of the footplate, and movement of the visual surrounding. Participants are tested in 6 conditions (20 s trials and three trials for each condition) (See Fig. 14): **Condition 1**: Eyes open, fixed footplate, fixed visual surround; **condition 2**: Eyes closed, fixed footplate, fixed visual surround; **condition 3**: In this condition, the participant stands with their eyes open; the visual surround is sway-referenced and thus visual information is compromised. In **condition 4**: the support surface is sway-referenced; therefore, somatosensory information is compromised for the participant. In **condition 5**: the participants stand on a sway-referenced support surface with closed eyes. Thus, balance is maintained through vestibular cues with visual cues removed and compromised somatosensory cues. In **condition 6**, the visual surround and support surface are both sway-referenced, identifying whether the participant relies on vestibular information when both visual and somatosensory cues are inaccurate. The first 3 SOT conditions are performed on a static support surface, while the last 3 SOT conditions use a dynamic support surface (impairing proprioceptive information). The SOT equilibrium scores represent the maximum anterior-posterior sway during each trial. A participant’s highest score (100) is indicative of no sway and lowest score (0) indicates fall during the trial.

#### Motor control test (MCT)

The MCT consists of 3 forward and 3 backward footplate translations that are graded in magnitude (small, medium and large). Medium magnitude translations were conducted and analyzed for each participant, and the support surface’s horizontal displacement during translation was scaled according to the participant’s height. Motor latency was evaluated as the time lapse (ms) between the support footplate translation and the participant’s active force response. This latency information is valuable for identifying motor system abnormalities^24^. Prolonged latencies imply dysfunction in the long-loop automatic motor system and may be associated with central or peripheral system lesions^40^.

#### Gross motor function measure-88

The GMFM is a standardized assessment and can measure the change in gross motor function over time in children. The GMFM has 88 items in 5 dimensions; A: Laying and rolling, B: Sitting, C: Crawling and Kneeling, D: Standing and E: Walking, running and jumping. Each item in each dimension is scored on a four-point Likert scale. The instrument has been validated in children with cerebral palsy ages 5 months to 16. A typical 5-year-old child without any motor disabilities can reach the maximum score of 100. GMFM has been recently suggested as an important tool for motor function evaluations in childhood neuronal ceriod lipofuscinosis(NCL)^41^.

## Data Availability

The datasets are not publicly available due to restrictions used under the license for the current study. They are available on reasonable request from the corresponding author.
